# The “Immunoscore” in rectal cancer: could we search quality beyond quantity of life?

**DOI:** 10.18632/oncotarget.28100

**Published:** 2022-01-05

**Authors:** Amos Kirilovsky, Carine El Sissy, Guy Zeitoun, Florence Marliot, Nacilla Haicheur, Christine Lagorce-Pagès, Julien Taieb, Mehdi Karoui, Petra Custers, Edina Dizdarevic, Soledad Iseas, Torben Frøstrup Hansen, Lars Henrik Jensen, Geerard Beets, Jean Pierre Gérard, Mireia Castillo-Martin, Nuno Figueiredo, Angelita Habr-Gama, Rodrigo Perez, Jérôme Galon, Franck Pagès

**Affiliations:** ^1^Laboratory of Integrative Cancer Immunology, INSERM, Paris, France; ^2^Equipe Labellisée Ligue Contre le Cancer, Paris, France; ^3^Centre de Recherche des Cordeliers, Sorbonne Université, Université de Paris, Paris, France; ^4^Immunomonitoring Platform, Laboratory of Immunology, Assistance Publique-Hôpitaux de Paris (AP-HP), Georges Pompidou European Hospital, Paris, France; ^5^Department of Pathology, AP-HP, Georges Pompidou European Hospital, Paris, France; ^6^Department of Gastroenterology and Gastrointestinal Oncology, AP-HP, Georges Pompidou European Hospital, Université de Paris, Paris, France; ^7^Department of Digestive Surgery, AP-HP, Georges Pompidou European Hospital, Université de Paris, Paris, France; ^8^Department of Surgery, Netherlands Cancer Institute – Antoni van Leeuwenhoek, Amsterdam, The Netherlands; ^9^GROW School for Oncology and Developmental Biology, Maastricht University, Maastricht, The Netherlands; ^10^Department of Oncology, Vejle Hospital, University Hospital of Southern Denmark, Vejle, Denmark; ^11^Danish Colorectal Cancer Center South, Vejle Hospital, Vejle, Denmark; ^12^Oncology Unit, Gastroenterology Hospital, Dr. Carlos Bonorino Udaondo, Ciudad Autónoma de Buenos Aires, Argentina; ^13^Department of Radiation Oncology, Centre Antoine Lacassagne, Nice Sophia-Antipolis University, Nice, France; ^14^Service of Pathology, Champalimaud Foundation Biobank (CFB)/Champalimaud Centre for the Unknown/Champalimaud Foundation, Lisbon, Portugal; ^15^Colorectal Surgery, Digestive Department, Champalimaud Foundation, Lisbon, Portugal; ^16^Colorectal Surgery, Lusiadas Hospital Lisboa, Lisbon, Portugal; ^17^Department of Colorectal Surgery, Angelita & Joaquim Gama Institute, São Paulo, Brazil; ^*^These authors contributed equally to this work

**Keywords:** rectal cancer, immunoscore, watch and wait, prognosis, radiochemotherapy

## Abstract

Because of the function and anatomical environment of the rectum, therapeutic strategies for local advanced rectal cancer (LARC) must deal with two challenging stressors that are a high-risk of local and distal recurrences and a high-risk of poor quality of life (QoL). Over the last three decades, advances in screening tests, therapies, and combined-modality treatment options and strategies have improved the prognosis of patients with LARC. However, owing to the heterogeneous nature of LARC and genetic status, the patient may not respond to a specific therapy and may be at increased risk of side-effects without the life-prolonging benefit. Indeed, each therapy can cause its own side-effects, which may worsen by a combination of treatments resulting in long-term poor QoL. In LARC, QoL has become even more essential with the increasing incidence of rectal cancer in young individuals. Herein, we analyzed the value of the Immunoscore-Biopsy (performed on tumor biopsy at diagnosis) in predicting outcomes, alone or in association with clinical and imaging data, for each therapy used in LARC.

## INTRODUCTION

About a third of colorectal cancer cases are located in the rectum, making rectal cancer the eight most common cancer worldwide and a major health issue [1]. Rectal cancer has distinct immune environment and gene expression profiles, fewer BRAF mutations, and less microsatellite instability compared to colon cancer [2–5]. Moreover, unlike colon cancer, it is associated with a worse prognosis in early-stage disease, but with longer survival in more advanced-stage [6]. The management of rectal cancer varies from that of colon cancer because of the increased risk of pelvic recurrence and a poorer overall survival (OS). Since the first description of radical surgical procedure, the history of rectal cancer treatments has been driven by the necessity to improve both oncological outcomes and quality of life (QoL), especially in patients with mid/low locally advanced (T3–T4, N0 or Tx, N1–2, M0) rectal cancer (LARC). These aims have however been complicated not only by the risk of local and distant recurrences, but also by the high-risk of bowel, urinary, and sexual dysfunctions due to a challenging anatomical environment. Improving the long-term QoL has become even more essential since rectal cancer is extending over a wider age range with the incidence on the rise among young adults in the USA and Europe expecting to reach a peak in a group of patients younger than 35 years within the next decade [1, 7].

During the last 30 years, breakthrough innovations as well as continuous evolution of technologies, techniques, and strategies have led to a shift from a surgical-dominated management to a multidisciplinary management of rectal cancer. Nowadays, combined-modality management strategies (including chemotherapy, radiation therapy, and surgery) significantly improve oncological outcomes by decreasing local recurrence and distant metastasis with a good therapeutic compliance. As a result, in appropriately treated patients, the 5 and 10-year OS rates are estimated to be 60% and 50%, respectively [8]. Moreover, due to significant downsizing of the tumor following neoadjuvant therapy, a complete pathologic response (pCR), associated with a low-risk of local and distant relapses, may be obtained [9]. The latter gave rise to the so-called “Watch-and-Wait” strategy [10], initially considered for patients with advanced disease and at high-risk for a definitive stoma following surgical resection, is now considered a very attractive alternative by patients and clinicians, even in the setting of early stage disease. The management of rectal cancer with organ-preserving strategies allows avoiding radical surgery, postoperative complications, and short-term poor QoL particularly due to stoma formation. However, each therapy has side-effects that can be worsened by the use of a combination of therapies resulting in poor long-term QoL [11].

In cancer treatment, patients are often required to consider trade-offs between QoL and length of life (LoL) [12]. This involves weighing the risks and benefits of treatment according to the patients’ concerns and expectations. As these multimodality treatment strategies become more common, but also more complex, clinicians and patients need the most accurate information before deciding which disease management pathway to follow because of the trade-off between QoL and LoL.

In this context, we herein review therapeutic strategies for mid/low LARC and discuss the potential of the “Immunoscore” performed on biopsies (IS_B_) obtained at the time of diagnosis to identify, alone or in association with other pertinent pretherapeutic markers, good or poor responders.

### Therapeutic management of mid/low LARC: a still ongoing debate

#### The standard of care

For decades, total mesorectal excision (TME) alone has been the cornerstone treatment for LARC [13]. After 2004, in locally advanced disease, neoadjuvant chemoradiotherapy (nCRT) with 5-fluorouracil (5-FU) was adopted as the standard of care on the basis of the German Rectal Cancer phase III clinical trial CAO/ORO/AIO-94 [14] and two confirmatory trials [15–17], assessing the superiority of nCRT over adjuvant CRT. Radiotherapy is delivered either as a short-course (SCRT, 25 Gy in 5 fractions) or as a long course (LCRT 45-50 Gy in 25 fractions) with concomitant chemotherapy. nCRT has led to a better QoL of patients suffering from rectal cancer by increasing sphincter sparing and a good local control of the disease, unfortunately without survival benefit [18–20].

#### Adjuvant chemotherapy

The use of adjuvant chemotherapy following nCRT and surgery to eradicate any micro-metastasis is still debatable [21–23]. The current National Comprehensive Cancer Network (NCCN) guidelines [24] recommend adjuvant chemotherapy following nCRT and radical surgery based on the published evidence from colon cancer studies [25, 26]. However, it is now known that clinical course and biology significantly differ between the colon and rectum. Therefore, rectal cancer should be considered and treated as a separate entity and extrapolation of the use of adjuvant chemotherapy from colon cancer studies should be avoided [27]. Among the five recent European trials (CHRONICLE, QUASAR, EORTC 22921, PROCTO-SCRIPT, I-CNR-RT) of stage II-III rectal cancer patients investigating the benefits of adjuvant chemotherapy after nCRT and surgery, in only one trial (QUASAR) a significant increase in survival in the adjuvant chemotherapy group was observed. Several meta-analysis have failed to detect difference in disease-free survival (DFS) and OS with adjuvant treatment compared to surveillance [28, 29], leaving the adjuvant treatment option an ongoing debate.

#### Total neoadjuvant therapy

In order to improve DFS and OS and optimize treatment of patients with LARC, several small trials have tested “total neoadjuvant therapy” (TNT), a new approach in which systemic induction chemotherapy precedes nCRT and resection [30–34]. In the Spanish GCR-3 phase II randomized trial, patients were randomized to receive CAPOX either before nCRT or after surgery. No differences in DFS, OS, and the cumulative incidence of local and distant relapse were observed and induction chemotherapy was associated with a better compliance, less toxicity, and better tolerance [35]. The results of the RAPIDO trial were recently published [36]. In the trial patients were either allocated to the experimental TNT group (short-course radiotherapy followed by 6 cycles of CAPOX or 9 cycles of FOLFOX4 followed by TME) or to the standards of care group (long course radiotherapy with concomitant oral capecitabine followed by TME with or without CAPOX or FOLFOX4-based adjuvant chemotherapy). The primary endpoint was 3-year disease-related treatment failure. The TNT experimental treatment decreased the rate of disease-related treatment failure compared to standard of care, mainly due to fewer distant metastases.

Although, the TNT strategy with induction chemotherapy is still under debate, it could be beneficial in the early prevention or eradication of micrometastases, inducing higher pCR, facilitating surgical resection, and promoting patient’s compliance to treatment as patients are more likely to complete the treatment schedule. Yet, a potential disadvantage to this intensive approach is the possibility to overtreat patients with low-risk stage II rectal cancer and to induce persistent neurotoxicity from oxaliplatin. NRG-GI002 (NCT02921256) is a phase II randomized trial that evaluates different TNT strategies (veliparib or pembrolizumab) in LARC and serves as a modular platform to assess novel sensitizers to neoadjuvant chemotherapy and/or CRT [37]. In a recent meta-analysis by Petrelli et al. [9], the addition of induction or consolidation chemotherapy to the standard nCRT resulted in a higher pCR rate. Large confirmatory trials with OS or DFS used as a surrogate endpoint for OS are needed before any recommendation is made in favor of TNT.

#### High dose CRT, brachytherapy, contact radiotherapy

At high enough dose, radiation has the potential to eradicate all cancer cells, but this strategy is limited by toxicity to the neighboring organs. There is a growing interest to improve the rate of clinical response to nCRT by intensification of pre-operative treatment, especially by different radiotherapy techniques [38].

One way of increasing radiation dose to the tumor is endorectal brachytherapy. This method uses Iridium-192 (Ir-192) in a remote after-loading system. A phase III trial showed that brachytherapy boost was feasible with no increase in toxicity [39]. The same Danish group assessed in a clinical trial the efficacy of increased radiation dose in LARC [40]: a radiation dose of 60 Gy plus an endorectal boost of 5 Gy to the tumor led to a clinical complete response (cCR) in 78% of patients, a higher rate than that reported in trials with the traditional radiation dose.

Another option to achieved radiotherapy dose escalation in LARC is contact X-ray therapy (CXRT) [41–43]. CXCRT in rectal cancer was popularized by Jean Papillon in Lyon (France) who used a portable 50-kV X-ray machine. This unit delivered a 50-kV X-ray beam with a source skin distance (SSD) of 4 cm and a dose rate of 20 Gy/min. Sun Myint et al. studied the impact of contact therapy dose escalation on organ preservation [44]. Organ preservation was achieved in 62% of patients (a higher rate than that reported by most of the watch-and-wait studies); 11% of patients presented local regrowth compared to 30% after classical external beam chemoradiotherapy. The CXCRT approach has the potential to reduce dose to normal tissue and reduce toxicity. It is used primarily in the adjuvant setting after local excision in patients for whom radical surgery is not indicated due to age or poor performance status.

#### On-going trials on resectable mid/low LARC: can radiotherapy be avoided?

Radiotherapy in the pelvic area is commonly associated with complications such as anastomotic leakage (combined to TME), sexual dysfunction, and fecal incontinence. Ongoing clinical trials are currently exploring how to avoid radiotherapy, with either preoperative systemic therapy or postoperative chemotherapy.

In the NORAD01-GRECCAR16 multicenter phase III trial of patients with primarily resectable LARC, preoperative systemic chemotherapy without pelvic irradiation is tested as an alternative to CRT [45]. The choice of modified FOLFIRINOX for preoperative chemotherapy is supported by the recent and consistent data on the safety and efficacy of this regimen from large phase III trial of patients with LARC [46]. The rationale of this trial is that the use of preoperative chemotherapy instead of CRT offers the potential benefits in terms of functional results and QoL in cancer survivors. Although, the non-inferiority of preoperative chemotherapy compared to CRT on oncologic outcome has to be validated. Once demonstrated, this could lead to a crucial change in clinical practice in a large subset of rectal cancer patients.

The phase II TME-FOLFOX study aims to evaluate the efficacy of upfront radical surgery with TME followed by adjuvant chemotherapy with folinic acid (or leucovorin), 5-FU, and oxaliplatin (FOLFOX) versus the current standard treatment in patients with surgically resectable LARC. The study investigates whether patients with cT_1-2_N_1_ or cT_3_N_0_ without circumferential resection margin (CRM) involvement and lateral lymph node metastasis as evaluated on preoperative high-resolution magnetic resonance imaging (MRI) may not require preoperative CRT [47]. If in resectable mid/low LARC, omission of CRT is non-inferior to the standard treatment; radiation-related toxicity could be avoided.

#### Complete clinical response and the watch-and-wait strategy

Neoadjuvant chemoradiation therapy can have a substantial effect on tumor cell death in rectal cancer; in about 20% of LARC patients, a pCR (no evidence of viable tumor cells are found in the surgical specimen) has been reported after nCRT [48, 49]. Given the excellent outcomes of these patients, in 2004, Habr-Gama and her group, from São Paulo in Brazil, published a first report where 71 patients with a complete clinical response (cCR) entered a strict surveillance program and avoided surgery with similar survival outcomes compared to patients with pCR after radical surgery [50]. The results from several single center studies have confirmed that accurately selected patients with cCR could benefit from this non-operative strategy [51–54]. Data from the International Watch & Wait Database (IWWD) study of the largest series of “watch-and-wait” patients with rectal cancer showed outstanding outcomes (the 5-year OS and DSS were 85% and 94%, respectively) [55]. However, the question on appropriate selection of clinical complete responder is still being evaluated [55–57]. Clinical (digital rectal examination) and endoscopic criteria of a cCR have been reported by Habr-Gama et al. in 2010 [58]. Maas et al. [59] suggested these findings as the most accurate clinical assessment methods to identify complete clinical responders. Moreover, they suggested that the addition of high-resolution MRI could improve the diagnostic performance.

While patients who achieve a complete clinical response are clearly the best candidates for the Watch-and-Wait strategy, patients with initial near complete response could also potentially avoid definitive surgical resection. Indeed, some of these latter patients may benefit from an extended waiting interval [60] or from additional local treatment strategies such as local excision or contact X-ray brachytherapy [44], as a form of organ-preserving strategies without radical TME. However, a subset of these patients will still require radical surgery. Current challenges include accurate selection of patients to avoid both overtreatment of patients that will benefit the best of organ-preservation strategies and undertreatment of patients that need TME.

Non-operative strategy continues to be an active and controversial area of investigation, since the determination of complete clinical responders to nCRT still requires a powerful and sufficiently robust tool for its clinical utility.

### Assessment of response to nCRT

With the current debate on the therapeutic management of LARC (nCRT, induction chemotherapy, high dose CRT, endorectal brachytherapy, CXCRT, the watch-and-wait strategy), there is a crucial need for biomarkers predictive of the quality of response to nCRT. The ability to predict which patients will truly benefit from the nCRT would result in improved patient stratification first by directing patients who are likely to achieve a good response to non-operative strategy and secondly by intensifying nCRT (e.g., the use of induction/consolidation chemotherapy, RT dose escalation) in patients unlikely to respond to standard treatment regimens.

However, there is a lack of effective methods to predict which patients with LARC would or would not respond to nCRT and have benefit from additional treatment strategies. Although a number of molecular biomarkers (in tumoral tissues or blood) have been proposed as predictors of response to nCRT, none of them has reached the clinic [61]. Several molecular biomarkers in tumoral tissues have been described to be associated with pathological response such as TP53 and KRAS. A meta-analysis results based on data from 30 published studies of 1830 patients with rectal cancer suggested that the wild-type p53 status is associated with pCR following nCRT [62].

Gene expression profiling of tumoral tissues in rectal cancer has the potential to identify gene signatures associated with good response to nCRT. Several gene expression signatures have been published [63–67]. In a recent review Izzotti et al. summarized the current knowledge on genetic and epigenetic biomarkers used as predictors of response following nCRT [68]. After an extensive review of the literature, only 19 mRNAs and 6 miRNAs were found to be expressed differentially among responders versus non-responders in two or more independent studies. However, none of these biomarkers appears to be predictive when examined alone, thereby, significant discrepancies across studies were reported. The authors suggest a miRNA signature as a predictor of response to nCRT. However, this would first need to be tested in retrospective and prospective studies.

The relationship between carcinoembryonic antigen (CEA) and response to nCRT has been well studied [69–72]. CEA levels, either prior to treatment or after nCRT and before radical surgery, was found to be a factor associated with tumor regression and pCR [73, 74].

A recent study evaluated the clinical utility of circulating tumor DNA (ctDNA), which is secreted from cancer cells into the peripheral blood, to predict responses to nCRT of patient with LARC [75]. Change in ctDNA was found an independent predictor of complete response to nCRT. Large confirmation studies are yet needed to assess the prognostic value of ctDNA.

To date, no predictive biomarker was robust enough to stratify patients according to their level of response to nCRT. The integration of diverse type of biomarkers including clinicopathological and imaging features would allow developing robust and cost-effective biomarkers facilitating a personalized treatment strategy for patients with rectal cancer, and improving selection of responders who might benefit from intensified treatment, and avoiding non-responders to receive an intensified treatment without the benefit of this treatment. Activation of host immune response plays an important role in the therapeutic effects of chemoradiation [76]. Several research groups have assessed the predictive and prognostics effects of tumor microenvironment in patients with rectal cancer treated by nCRT [77–80]. For instance, the association between PDL-1 expression and response to nCRT has been intensively studied; however there was no consistency of the result seen across studies. This might be the effect of technical or biological issues, different thresholds for PDL-1 detection, and the variability of tissue preparation. Therefore, a standardized evaluation of the tumor microenvironment is crucial.

### Immunoscore

#### Conceptual bases and development in colon cancer

Cancer natural history involves interactions between tumor and host defense mechanisms [81]. The ‘cancer immunosurveillance’, a concept where the immune system can recognize and eliminate primary developing tumors is now well described with a considerable amount of data from animal models and human patients [82]. When the tumor elimination is incomplete, a temporary state of equilibrium occurs. The selective pressure exerted by the immune cells induces a selection of tumor cell variants that leads to the escape phase. At that point, the immune system is no longer able to contain tumor growth leading to clinically detectable malignant tumors [82].

Although solid tumors are able to escape from the immune system, many immune cells are present within the tumor glands, in the surrounding stroma, within the invasive margin, and in newly formed tertiary lymphoid islets located in the tumor vicinity [83]. As the immune infiltration in tumors is heterogeneous, we hypothesized that analysis of each tumor region could provide information on the tumor pathophysiology and possibly on prognosis. We thus measured the densities of immune cells and their distribution in the tumor core (CT) and the invasive margin (IM) of colorectal cancers and found that the immune ‘contexture’, defined as the type, functional orientation, density, and location of adaptive immune cells within distinct tumor regions [84, 85], appears to be the strongest prognostic factor for survival and tumor dissemination.

We then derived a simple test named Immunoscore (IS) (http://www.Immunoscore.org) to facilitate the transfer of this discovery to the clinic [86–88]. This tool is based on the numeration of two lymphocyte populations, T CD3+ cells and T CD8+ cytotoxic cells, in the CT and in IM regions. The analysis of IS analytical performance characteristics showed that it is a robust, reproducible, quantitative, and standardized immune assay [89].

An international Immunoscore consortium led by the Society for Immunotherapy of Cancer (SITC) confirmed that the consensus IS tested in 3539 stage I–III colon cancer patients holds a prognostic value superior to that of the AJCC/UICC-TNM staging system [90]. The consensus IS, is the first internationally validated standardized digital-pathology-based assay to quantify the immune infiltrate [86–88] and the first biomarker recommended by academic institutions quantifying the immune infiltrate in the tumor for a prognostic purpose (the 2020 ESMO guidelines [91]; thereby quantification of the immune infiltrate in the tumor has now been added to the 5th edition of WHO Digestive System Tumours book).

#### IS predicts adjuvant chemotherapy response in colon cancer

A still-open question is whether the tumor immune status might further determine the extent of response to chemotherapy [87, 92, 93]. An immune-related gene signatures predicting the outcome of neoadjuvant chemotherapy has been revealed in breast carcinoma [94]. The prognostic value of IS and its association with the effect of adjuvant chemotherapy have been recently investigated in two studies of stage III colon cancer [99, 100]. In the international Immunoscore study of the pre-defined consensus IS reported by Mlecnik et al., 763 patients with AJCC/UICC-TNM stage III CC were evaluated [95]. In the study, only patients with an Intermediate or High-IS responded to chemotherapy and had prolonged survival versus those without chemotherapy (HR = 0.42; 95% CI, 0.25 to 0.71; *P* = 0.0011). Contrarily, patients with Low-IS did not significantly benefit from chemotherapy treatment regardless of the estimated risk level (high-risk [*P* = 0.12], low-risk [*P* = 0.17]). The second study, the Immunoscore-IDEA France trial conducted in collaboration with PRODIGE, a digestive oncology intergroup gathering the GERCOR, the FFCD, and UNICANCER organizations [96], investigated the ability of the IS to predict response to adjuvant chemotherapy in 1062 stage III colon cancer patients. This trial was a part of The International Duration Evaluation of Adjuvant Chemotherapy (IDEA) collaboration phase 3 trial that prospectively aimed to evaluated the noninferiority of 3 versus 6 months of adjuvant therapy with either FOLFOX or CAPOX in patients with resected stage III colon cancer [97]. For FOLFOX-treated patients (91.6% of the cohort), a statistically significant interaction was observed for the predictive value of IS for treatment duration (3 versus 6 months) in terms of DFS. Intermediate or High-IS significantly predicted benefit of 6 months treatment (HR = 0.53; 95% CI, 0.37 to 0.75; Log-rank *P* = 0.0004), including clinical low (T_1-3_ N_1_) and high-risk (T_4_ or N_2_) patients (all *P* < 0.001). Conversely, patients with Low-Immunoscore (46.4%) did not exhibit significant benefit from the 6-month FOLFOX versus 3-month. These patients appeared to be doubly penalized by an increased risk of recurrence and the lack of benefit from longer duration of treatment [98].

Component drugs in the FOLFOX regimen include 5-FU, which may partially deplete or transiently inactivate inhibitory immune cells [99], and oxaliplatin, a chemotherapeutic agents eliciting bona fide immunogenic cell death [100]. Chemotherapy activity could thus in a part be mediated by an immune anti-tumoral response that might eliminate disseminated tumoral cells after cancer resection.

#### IS and rectal cancer

In 1986, Jass [101] demonstrated for the first time that the high lymphocyte density evaluated on histological sections in the IM of rectal tumors was the only variable retained in a multivariate prognostic model alongside with the tumor, nodes, metastasis (TNM) classification. This observation was further confirmed in other studies [102, 103]. Recently, we have also validated this result in a cohort of 111 patients with rectal cancer who did not receive nCRT [104]. The IS was assessed through the CD3+ and CD8+ T cell densities quantification in the CT and IM regions by immunohistochemical-based tissue microarray analyses with image analysis software. A significant association between IS and differences in DFS and OS (HR = 1.81 and 1.72, respectively; all *P* < 0.005) was observed. The IS was also a stronger prognostic factor than the TNM staging in predicting recurrence and survival in cox multivariate analysis (all *P* < 0.001). Interestingly, among a small cohort of 33 patients who would be eligible to nCRT at present, the CD3+ and CD8+ cell densities were decreased in those who experienced relapse [104].

#### Immunoscore biopsy (IS_B_): a derived immunoscore

Preliminary studies in rectal cancer have suggested that the natural immune reaction of tumors could be evaluated on biopsies [104–106], the only sample material available before nCRT. Indeed, nCRT induces architectural and histological changes on the surgical specimen that prevent the assessment of a classic IS in LARC patients who underwent nCRT. Therefore, diagnostic biopsies are the only way of retrieving tumoral tissue prior to nCRT. A derivation of the IS performed on initial biopsies (IS_B_) before nCRT allows to evaluate the quality of the initial immune response in the tumor and its potential influence on both the degree of response to nCRT and the clinical outcome [107].

**Figure 1:**
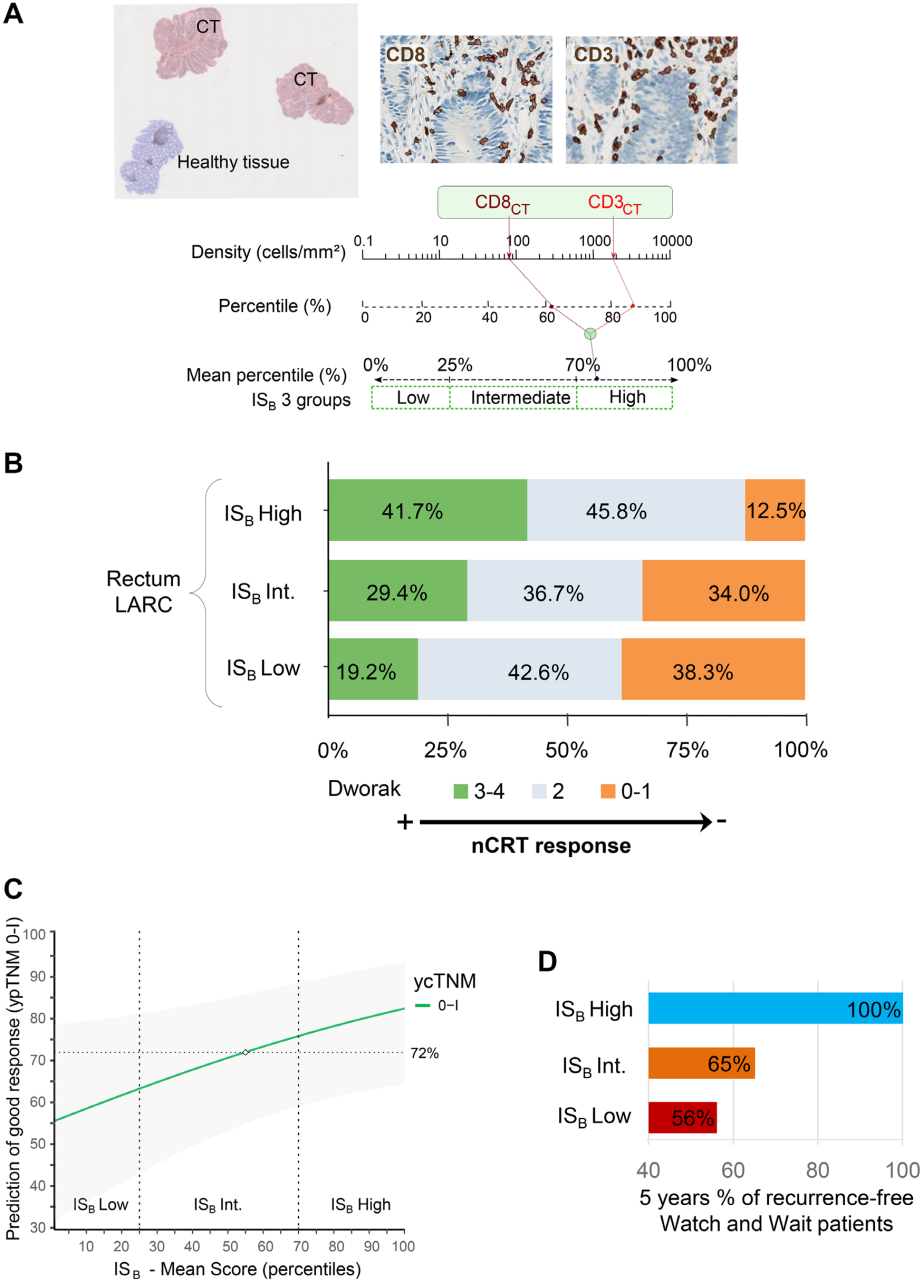
(**A**) Left: Representative image of rectal biopsies with tumor region (pink) and normal tissue (blue) or dysplasia excluded from the analysis (blue). Right: CD3+ and CD8+ T cells automatic detection. Bottom: Chart illustrating the IS_B_ calculation method: Densities of CD3+ and CD8+ T cells in the tumor are converted into percentile. The mean percentile of the densities are then calculated to generate IS_B_ percentile value, where IS_B_ low, intermediate and high subgroups are reflected by 0–25%, >25–70% and >70–100% percentile respectively. (**B**) The frequency of patients in each IS_B_ groups, according to the Dworak classification in locally advanced rectal cancer patients. (**C**) Prediction of pathologic response (ypTNM 0–1) based on IS_B_ mean score and the ycTNM classification ycTNM 0–I. (**D**) Percentage of recurrence-free in Watch and Wait patients after 5 years of treatment.

To determine IS_B_, diagnostic biopsies were immunostained to detect and quantify CD3+ and CD8+ T cells in the tumor area by digital pathology (Figure 1A). Two independent cohorts (*n*_1_ = 131, *n*_2_ = 118) of LARC patients treated with nCRT followed by radical surgery and one multicentric cohort of patients (*n* = 73) treated with the watch-and-wait strategy were investigated.

First, the IS_B_ prognostic performance was tested in two independent cohorts treated by surgery. In the first cohort, patients with IS_B_ High were at low risk of relapse, with the 5-year DFS of 91.1% (95% CI, 82.0 to 100) versus 65.8% (95% CI, 49.8 to 86.9) in patients with IS_B_ Low. These results were confirmed in a second independent cohort [*n*_2_ = 118; *P*_tft_ = 0.021; HR_(High vs. Low)_ = 0.25; 95% CI, 0.07 to 0.86).

Next, the correlation between the degree of histologic response to nCRT and IS_B_ was evaluated. Several classification can assess the degree of histologic response: i/the NAR score [108]: calculated using the equation [5pN-3(cT-pT) + 12]^2^/9.61, and classified as low (<8), intermediate (8–16), and high (>16), ii/the Dworak classification [109] defined as complete (Dworak 4), near complete (Dworak 3), moderate (Dworak 2), minimal (Dworak 1), and no regression (Dworak 0) (Figure 1B), and iii/ypTNM staging i.e., the postsurgical pathologic T and N evaluation. IS_B_ was correlated to nCRT response assessed by these three classifications (*P* < 0.001). As an example, IS_B_ High patients were not found in the non-responders Dworak 0 group. IS_B_ combined to imaging post-neoadjuvant treatment increased the accuracy of prediction of histologic good responders (ypTNM 0–I versus ycTNM) (Figure 1C). This information is of particular importance since (i) the accuracy of imaging to predict the complete response post-nCRT is not satisfactory; only 25% to 50% of the patients have a real histologic complete response (i.e., no residual tumor), and (ii) imaging is the gold standard in clinical practice to select patients’ eligible to a preservative strategy, and currently, no biomarker exists to personalize treatment.

The clinical utility of the composite biomarker (imaging + IS_B_) was tested within a cohort of watch-and-wait patients (*n* = 73) with post-nCRT cCR (ycTNM 0). Very interestingly, no evidence of relapse was observed during the follow-up period in IS_B_ High patients (23% of the cohort) (Figure 1D). These results suggested that IS_B_ would be a useful biomarker to select patients eligible for the watch-and-wait strategy, hence preserving their rectum.

#### Potential applications of IS_B_ and perspectives

The therapeutic multidisciplinary management of LARC (TNT, organ preserving strategies, etc.,) is receiving a growing interest among colorectal cancer specialists.

Although our first published results [107] still need large scale validation in retrospective cohorts, they suggested that IS_B_ could be used to predict tumor response after nCRT, restage local disease after nCRT, and predict clinical outcome. Indeed, one of today’s challenges in oncology is to predict whether LARC patients will achieve a (near)-complete response prior to neoadjuvant treatment. Thus, IS_B_ could be helpful in identifying bad responders who would benefit from accelerated treatment (a radiation boost) and in turns achieve complete response. Consequently, this could facilitate a personalized treatment approach for patients with rectal cancer and help to completely avoid ineffective treatment in non-responders.

Furthermore, considering the recent result of the ancillary trial evaluating the IS in the IDEA French cohort [96], the prognostic and predictive value of IS_B_ in rectal cancer patients treated by new therapeutic approaches such as adjuvant therapy [24], TNT, nCRT associated with adjuvant chemotherapy [110], or high dose nCRT [40] need to be evaluated. Certainly, IS_B_ could be a good predictive marker to select patients that would benefit from these intensive treatment strategies. Given the result of the IS study in the IDEA French cohort, rectal cancer patients with low IS_B_ might less or not benefit from adjuvant treatment.

The extensive and pioneering work of Prof Habr Gama in Brazil is presently considered as a reference for the use CRT and external beam radiation therapy boost followed by the watch-and-wait strategy that could safely replace surgery in more than 20% of rectal cancers. Still, in 25% of the patients managed by “watch-and-wait” local regrowth occurs [55]. Yet, no clinical, of biological biomarkers are currently accurate enough to stratify patients that could safely benefit from this strategy.

The IWWD study [55] aims to describe the outcome of watch-and-wait strategy in a large-scale registry. Confirming our first result in a large, multicentric, international cohort could strengthen the potential role of the immune assessment in diagnostic biopsies (IS_B_) in the selected LARC patients who could safely be followed by the watch-and-wait strategy.

IS_B_ is being evaluated in the OPERA ongoing ancillary study (NCT02505750). The trial aims to determine whether external beam radiation therapy versus endocavitary radiation therapy with contact X-ray brachytherapy after standard treatment with nCRT increases the chance of rectum and anus preservation in LARC. The evaluation of IS_B_ in such context will be of great interest to optimal patients’ selection.

## CONCLUSIONS

The IS, initially developed to predict the survival benefit of radical surgery in tumor samples, has recently proven to have predictive value for adjuvant chemotherapy response. However, many tumors including rectal cancers are now being treated by combined therapies where surgery may even be avoided, challenging the initial Immunoscore. We have thus developed an adapted-Immunoscore –IS_B_– that evaluates the natural immune reaction against a tumor on the only tumor material available before any therapy: tumor biopsies. This new biomarker should open new perspectives in personalized therapy especially given consistent data suggesting its capacity to predict the benefit of radiotherapy and chemotherapy, although prospective trials are needed to confirm this observation.
